# Microbiome and Autoimmune Uveitis

**DOI:** 10.3389/fimmu.2019.00232

**Published:** 2019-02-19

**Authors:** Reiko Horai, Rachel R. Caspi

**Affiliations:** Laboratory of Immunology, National Eye Institute, National Institutes of Health, Bethesda, MD, United States

**Keywords:** autoimmune uveitis, autoreactive T cells, commensal microbiota, gut, retina

## Abstract

Commensal microbes affect all aspects of immune development and homeostasis in health and disease. Increasing evidence points to the notion that the gut commensals impact not only intestinal diseases but also diseases in tissues distant from the gut. Autoimmune or non-infectious uveitis is a sight-threatening intraocular inflammation that affects the neuroretina. It is strongly T cell driven, but the precise causative mechanisms are not fully understood. We and others observed that depletion of gut microbiota in animal models of uveitis attenuated disease. Using a spontaneous model of the disease, we questioned how retina-specific uveitogenic T cells are primed when their cognate antigens are sequestered within the immune privileged eye. The data suggested that gut commensals provide a signal directly through the retina-specific T cell receptor and cause these autoreactive T cells to trigger uveitis. This activation of retina-specific T cells in the gut appears to be independent of the endogenous retinal antigen. Rather, the findings point to the notion that gut microbiota may mimic retinal antigen(s), however, the actual mimic has not yet been identified. Microbiota may also serve as an “adjuvant” providing innate signals that amplify and direct the host immune response for development of uveitis. In contrast, spontaneous uveitis that develops in AIRE^−/−^ mice appears to be independent of gut microbiota. To date, available data on human microbiota in association with uveitis are very limited and causative relationships are difficult to establish. This review will summarize the current knowledge on the role of microbiome in uveitis and its underlying mechanisms, and discuss unresolved questions and issues in an attempt to explore the concept of gut-retina axis.

## Introduction

Uveitis is one of the leading causes of blindness in the developed world and is responsible for 10–15% of severe visual handicap (1, 2). The disease affects working age population and has a significant impact on public health. Autoimmune (non-infectious) uveitis is a group of intraocular inflammatory diseases which target the neuroretina, where the light signal is converted into neural signals and subsequently sent to the brain and thus is considered as a part of the central nervous system (CNS). The disease can be part of a systemic autoimmune syndrome involving organs other than the eye, such as systemic sarcoidosis, Behçet's disease and Vogt-Koyanagi-Harada disease. In other cases, the eye may be the only target, such as in birdshot retinochoroidopathy, sympathetic ophthalmia, and idiopathic uveitis. Patients suffering from autoimmune uveitis often show detectable memory responses to unique retinal proteins, such as retinal arrestin and interphotoreceptor retinoid binding protein (IRBP), which are expressed in photoreceptor cells and participate in the visual process. Different uveitic diseases have been associated with particular HLA haplotypes (e.g., A29, B27, B51, DR4, DQ4), supporting the autoimmune nature of this condition (2). Although anecdotal evidence suggested that some forms of uveitis may be associated with systemic microbial infections, our understanding of disease etiology, driving mechanisms and treatment options are still limited. In the past several years, a number of publications indicated a causative role of gut microbiota in development of autoimmune diseases. Information in humans and animal models is accumulating, but is still sparse compared to those on other autoimmune syndromes affecting the brain or peripheral organs.

In this report, we will review evidence that gut microbiota can act as a trigger to activate autoreactive lymphocytes specific for the neuroretina through a process involving antigenic mimicry and microbial adjuvant effects, using mouse models of autoimmune uveitis. A role for microbial metabolites in regulation of uveitic disease has also been reported. However, a uveitis model has been reported that does not appear to be affected by commensal microbiota. Based on currently available evidence, we will discuss whether an immune-driven gut-retina axis, similar to the gut-brain axis, can be conceptualized.

## Mouse Models of Autoimmune Uveitis

Animal models of uveitis have been instrumental for our understanding of the pathogenesis of human uveitis. Mouse models, where various mutants are available thanks to the advanced technologies in genetic manipulation, have been particularly informative. The classical uveitis model, experimental autoimmune uveitis (EAU) is induced by active immunization with the retinal protein IRBP emulsified in complete Freund's adjuvant (CFA), a mixture of mineral oil with heat-killed *Mycobacterium tuberculosis* (MTB). Pertussis toxin is given as an additional stimulus in some strains of mice (e.g., C57BL/6) to facilitate disease induction. Susceptibility to disease is strain-dependent, and the immunization regimen is adjusted accordingly in terms of antigen and adjuvant dose. A particularly susceptible strain, which does not require pertussis toxin, is B10.RIII.

Co-administration of the bacterial adjuvant is required to activate innate immune cells and to create a proinflammatory milieu, that would subsequently induce adaptive immune responses and trigger the autoimmune effector pathways (3). However, unlike the experimental disease, most cases of human autoimmune uveitis cannot be directly connected to an exposure of the immune system to ocular antigens, which in the healthy eye are sequestered behind a tight blood-retinal barrier. This is a paradox because retinal antigens are not expressed in the periphery, but retina-specific T cells circulating in the periphery must be activated to be able to enter the eye and drive pathology. This situation raises a fundamental question where and how autoreactive T cells that can recognize retinal antigens and trigger uveitis first become activated.

**Figure 1:**
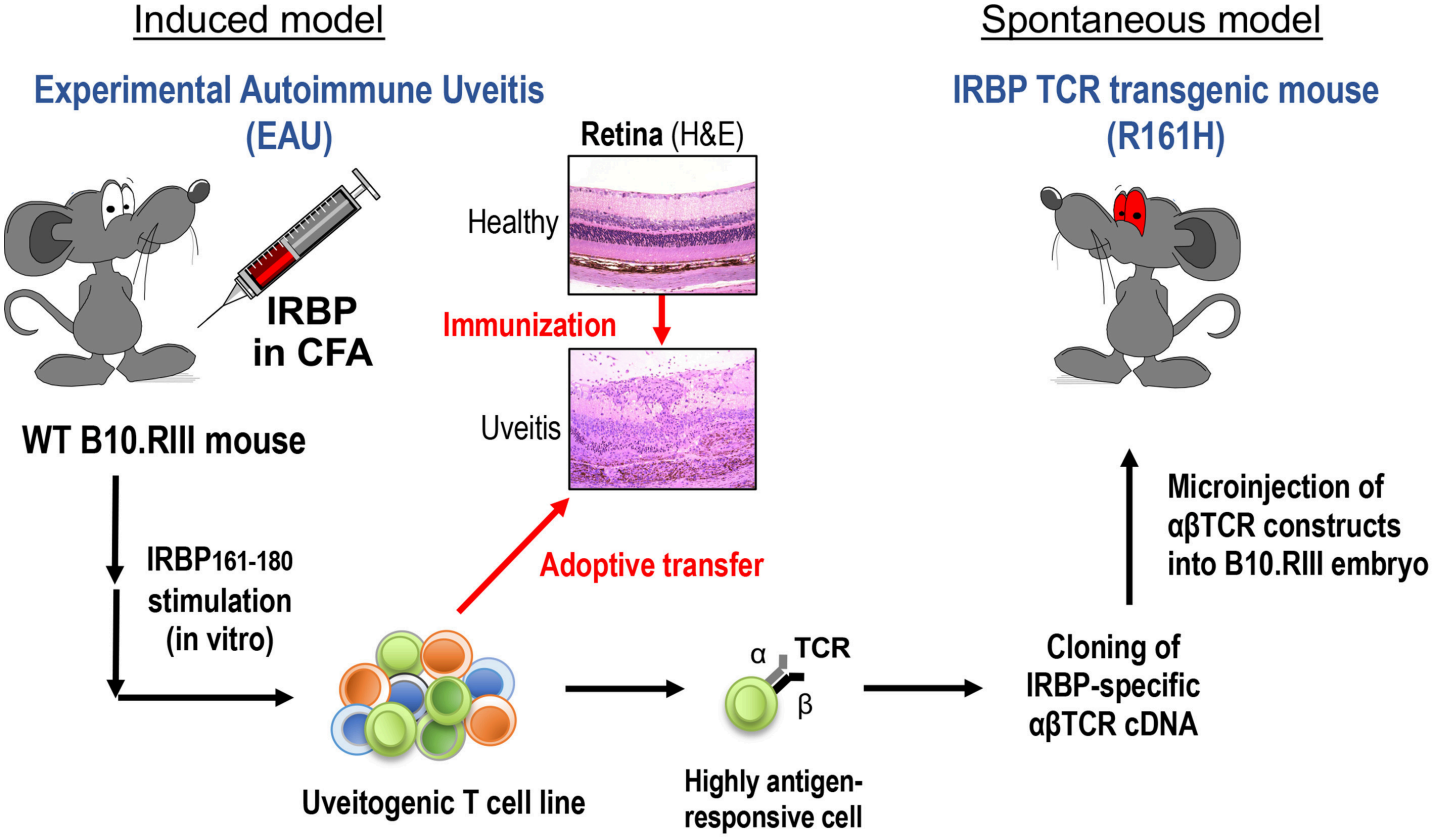
Induced and spontaneous models of autoimmune uveitis. Experimental Autoimmune Uveitis (EAU) is induced by active immunization of WT B10.RIII mice with the retinal autoantigen IRBP in complete Freund's adjuvant (CFA). Histology pictures show healthy and uveitic retina (H&E). A uveitogenic T cell line was established from draining LN cells of EAU-induced WT mice by several rounds of *in vitro* activation with uveitogenic peptide IRBP161-180. This cell line is highly pathogenic when adoptively transferred to naïve WT mice. The most highly antigen-responsive TCRαβ cloned from this cell line was used for generation of IRBP-specific TCR transgenic mice. A TCR transgenic line, R161H, develops spontaneous uveitis around weaning age.

To study natural triggers of autoimmune uveitis it is necessary to use spontaneous models of disease, as in the induced model, the trigger (uveitogenic immunization with retinal antigen) is provided by the investigator. We also need “amplified” models, so that the incidence is sufficiently high to be able to be studied in the laboratory. To satisfy these requirements, we developed a spontaneous uveitis model in T cell receptor (TCR) transgenic mice specific for a retinal protein (Figure 1).

The spontaneously uveitic R161H mice express an IRBP-specific TCR on the uveitis-susceptible B10.RIII background (4). R161H mice have an expanded peripheral population of CD4^+^ T cells (20–30%) specific for IRBP_161−180_ peptide, which is the major epitope of IRBP for the H-2^r^ haplotype expressed by B10.RIII mice. With this high precursor frequency of autoreactive T cells, R161H mice spontaneously develop autoimmune uveitis characterized by ocular inflammation, with lymphocytes and leukocytes infiltration and photoreceptor destruction, similar to the immunization-induced EAU model. First signs of disease are detected around weaning age, and the incidence reaches 100% by 2 months of age (4). This R161H model thus serves as a reproducible and robust model of spontaneous autoimmune uveitis. Using this model, we sought to ask whether commensal microbiota can serve as a microbial part of trigger in the development of uveitis, as will be described ahead.

## Commensal Microbiota as a Trigger of Uveitis

Commensal microbes colonize all exposed surfaces of the human body and outnumber our own cells by at least 10-fold. The gut is the most densely populated organ estimated to contain ~100 trillion commensal bacteria. In the past decade, dependence on gut commensals of inflammatory and autoimmune diseases in sites distal from the gut has been noted, but the mechanisms remained obscure. Since a large proportion of lymphocytes are found in the gut, we hypothesized that autoreactive cells might receive an activating trigger while passing through the gut. For the eye in particular, an activation step in the periphery seems crucial, because the antigens that are targeted in disease are sequestered in the (initially healthy) eye, and retina-specific lymphocytes must be in an activated state to be able to breach the blood-retinal barrier.

**Figure 2:**
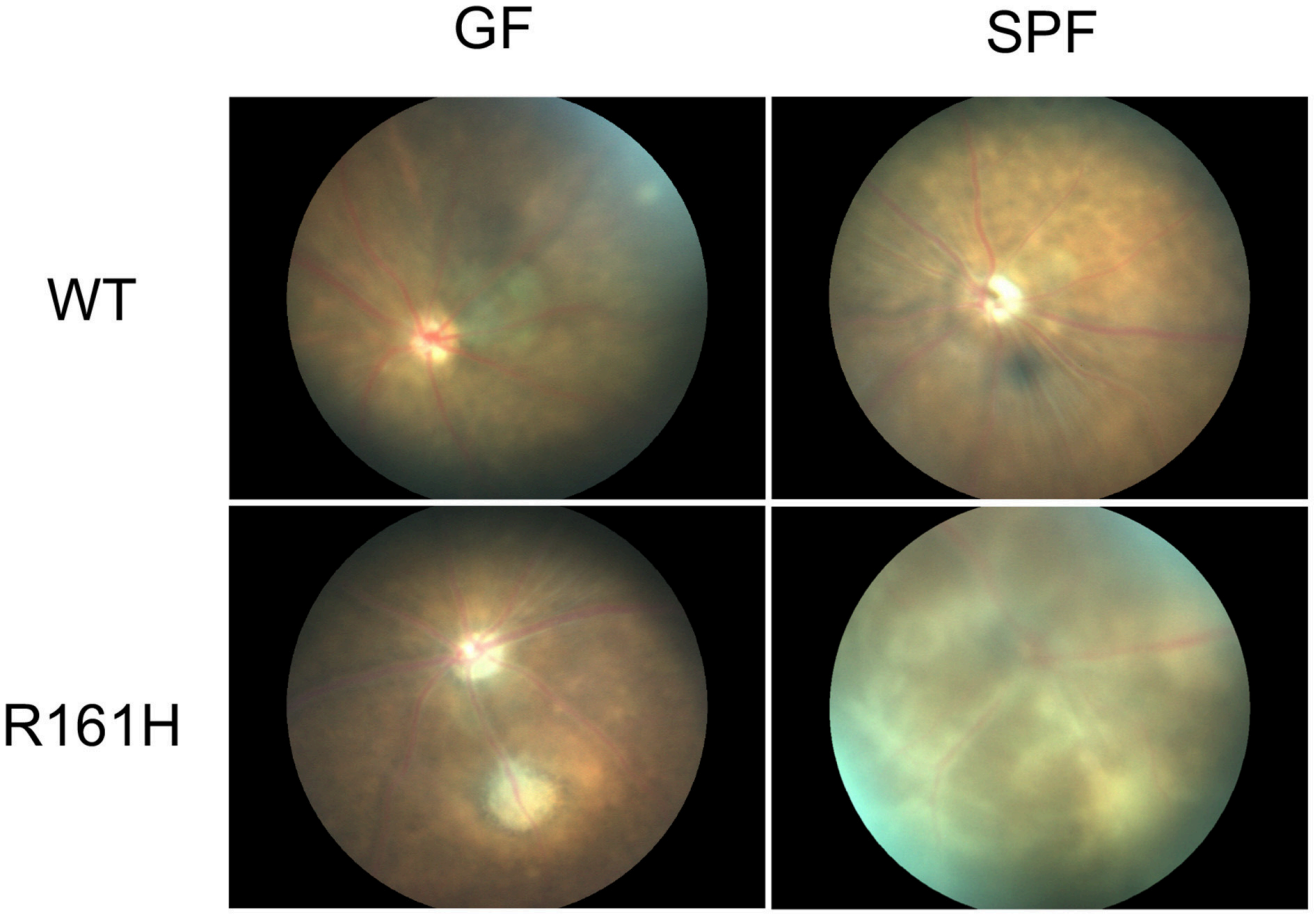
Fundus images of R161H mice under germ-free (GF) or specific pathogen-free (SPF) conditions. Fundus images were taken by Micron II. WT mice (SPF or GF) do not develop spontaneous uveitis and show normal fundus. SPF R161H mice develop moderate to severe uveitis and inflammation peaks between 2–3 months of age. Diffuse lesions and inflammation along the vessels and away from the vessels are present. GF R161H mice develop milder uveitis with focal inflammation/lesions. Photos were taken at 11 weeks old except for GF WT mice at 7 weeks old.

To examine this hypothesis, we used the spontaneous uveitis model in R161H mice to study the role of microbiota in triggering autoimmune uveitis (5, 6). A number of observations supported the interpretation that the antigen-specific activation of retina-specific cells occurs in the gut, and is dependent on gut microbiome. Activation of uveitis-relevant T cells was apparent in the intestinal lamina propria (LP) as early as 17 days of age, before onset of clinical uveitis. Depletion of commensal microbiota in R161H mice, by a cocktail of oral broad-spectrum antibiotics (ampicillin, metronidazole, neomycin, and vancomycin) given from before birth, resulted in significant attenuation of spontaneous disease, which was recapitulated in R161H mice reared under the germ-free (GF) conditions (Figure 2). Disease development was associated with increased populations of Th17 cells in the intestinal LP and in antibiotic-treated or GF R161H mice, these Th17 cells were significantly reduced. Co-housing GF R161H mice with SPF mice restored disease development (5). These results strongly supported the notion that commensal microbiota contributes to the development of spontaneous uveitis. The relative contributions of the TCR signal, and the likely contribution of non-specific effects, resulting from microbial “adjuvant” effects will be discussed ahead. It is important to add that, even under GF conditions, R161H mice over time will develop uveitis, albeit with reduced scores compared to SPF conditions. Therefore, microbial exposure is an important, but not the only, trigger for disease development in this model.

Nakamura et al. (7) reported that commensals also modulate uveitis in the immunization-induced EAU model. They used IRBP/CFA immunization of wild type (WT) B10.RIII mice, with a normal polyclonal T cell repertoire (7). Treatment starting 1 week before immunization with the same quadruple oral broad-spectrum antibiotic, that reduced development of uveitis in R161H mice, also reduced development of the immunization-induced disease. Confirmatory observations were reported in C57BL/6 mice under GF conditions (8). However, the effects of the microbiome on the spontaneous vs. induced uveitis models likely stem from distinct mechanisms. First and foremost, the antigenic and adjuvant triggers in the EAU model are provided by immunization with IRBP in CFA, whereas in spontaneous uveitis the trigger(s) are endogenous. Therefore, in contrast to spontaneous uveitis, microbiota can only have a modulatory, but not a causative role in induced EAU. Of note, an increase in Foxp3^+^ regulatory T cells (Tregs) were present both in lymphoid tissues (eye-draining lymph nodes and gut draining lymph nodes and LP) and in the eye of EAU-challenged mice that were treated with antibiotics (7). Single treatment of metronidazole or vancomycin (but not ampicillin or neomycin) was effective in decreasing clinical scores of EAU and inducing Tregs in LP. This approach might allow to narrow down the microbes that may have a promoting role in uveitis in this model. In contrast, however, no effects on Tregs were seen to be associated with antibiotic treatment and all 4 antibiotics were required to achieve protection in spontaneous uveitis (raising the possibility diverse classes of bacteria can promote disease) (6).

Complicating the picture, the immunization process itself appears to affect the gut microbiome. Metagenomic analysis by 16S rRNA sequencing revealed differences in microbiota composition between EAU-challenged mice and non-immunized (naïve) control mice that became significant 3 weeks after immunization (9). These findings suggest that host immune responses induced by immunization may modulate the intestinal microbiota, however, it is unclear whether this is due to antigen-specific effects or to the robust immune activation by CFA. Table 1 summarizes the differences in the effects of gut commensals on the spontaneous vs. the induced models of uveitis.

**Table 1:** Comparison of the effects of microbiota on disease and related immune responses in the spontaneous vs. induced uveitis models in B10.RIII mice.

**Effect**	**Spontaneous uveitis in R161H mice (5, 6)**	**EAU model induced by immunization with IRBP/CFA (7, 9)**
Prevention of disease by antibiotic treatment	Yes	Yes
Duration of protective effect	Prolonged	Temporary^*^
Treg induction during treatment	No	Yes (antibiotic driven)
Single antibiotics effective?	No—all 4 antibiotics needed	Yes (metronidazole or vancomycin)
Differences in gut microbiota between healthy and uveitis	None detected^*^	Yes

* *Supported by our unpublished data*.

## A Spontaneous Autoimmune Uveitis Model in Which Commensal Microbiota is Dispensable

Before discussing further mechanisms of how gut microbiota contributes to promoting uveitis, it should be useful to mention that there is an example of autoimmune uveitis model that is not dependent on microbiota. The AIRE protein is a transcription factor that is expressed in the medullary thymic epithelial cells and controls the promiscuous expression of tissue-specific antigens, including retinal proteins, thus it has a critical role in the process of thymic negative selection of autoreactive T cells (central tolerance). Loss of AIRE function in humans leads to the rare autosomal recessive disorder autoimmune polyendocrine syndrome type I (APS-1), which is characterized by multi-organ autoimmunity and susceptibility to infectious diseases. The *AIRE* gene knockout (*AIRE*^−/−^) mice develop multi-organ autoimmune diseases, including uveitis, that closely resemble human disease (10, 11). The manifestation of autoimmunity in *AIRE*^−/−^ mice depends largely on their genetic background and the presence of CD4^+^ T cells. Although thymic selection of T cells to retinal arrestin and other antigens controlled by AIRE is compromised in *AIRE*^−/−^ mice, IRBP is the only antigen recognized by these mice as pathogenic (12). *AIRE*^−/−^ mice on the C57BL/6 background or the NOD background both manifest autoimmune disorders, but the disease phenotype including uveitis is more severe in these mice on the NOD background (12). B6.*AIRE*^−/−^ mice were backcrossed onto B10.RIII in our lab to compare the pathology of spontaneous uveitis with R161H mice. B10.RIII *AIRE*^−/−^ mice reproducibly develop spontaneous disease at 5–6 weeks of age, but less aggressive ocular inflammation than R161H mice (13). GF *AIRE*^−/−^ mice were examined on the NOD background for the contribution of the commensal microbiota. Gray et al. (14) reported that immune cell infiltration of the retina as well as other tissues including lung, pancreas and stomach, was similar in *AIRE*^−/−^ mice under SPF and GF conditions. This suggests that autoimmunity caused by AIRE mutations is not dependent on the presence of commensal microbiota.

More recently, Proekt et al. (15) reported that a dominant-negative AIRE allele (G228W point mutation), with only partial penetrance, resulted in high incidence of uveitis when combined with LYN-deficiency (which by itself leads to a lupus-like disease). The findings support the notion of multiple gene defects combining for an overtly autoimmune phenotype. Interestingly, the eye was the only organ that appeared to be selectively affected in the double mutant mice. In line with the results in GF NOD.*AIRE*^−/−^ mice, the *AIRE*^*GW*/+^ *LYN*^−/−^ model showed that broad-spectrum antibiotics did not affect the incidence of spontaneous uveitis.

Because development of spontaneous uveitis in all three models: R161H, *AIRE*^−/−^ and *AIRE*^*GW*/+^ *LYN*^−/−^, is due to an increased frequency of retina-specific T cells, the discrepancy in terms of their dependence on endogenous microbiome appears puzzling. However, a fundamental difference is that, whereas the *AIRE*^−/−^ and *AIRE*^*GW*/+^ *LYN*^−/−^ mice have a diverse and high-affinity retina-specific T cell repertoire due to a failure of thymic negative selection, R161H mice express a single TCR specificity of relatively low affinity, originating from a post-thymic T cell that had been positively selected in an IRBP-expressing thymus. It is therefore conceivable that numerous endogenous or food-derived mimics may be able to trigger the high affinity retina-specific TCRs of *AIRE*^−/−^ and *AIRE*^*GW*/+^ *LYN*^−/−^ mice, explaining lack of dependence on microbiota. The various uveitis models and the main mechanisms that appear dominant in triggering disease are summarized in Table 2.

**Table 2:** Animal studies in gut microbiota and uveitis.

**Animal model**	**Intervention**	**Effect on disease**	**Resulting immune responses in the eye and/or the intestine**	**Reference**
R161H (B10.RIII)	Broad-spectrum antibiotics^a^, GF	Decreased	Reduced activated T cells in the gut, due to the lack of direct signaling via retina-specific R161H TCR	(5)
B10.RIII EAU	Broad-spectrum antibiotics^a^	Decreased	Increased Treg in the colon and eye	(7)
C57BL/6 EAU	Broad-spectrum antibiotics^b^, GF	Decreased	Reduced T cell infiltration in the retina	(8)
C57BL/6 EAU	SCFA (propionate)	Decreased	Induction of Treg in lamina propria and LN, and reduction of Th1 or Th17	(9)
C57BL/6 EAU	Probiotics mix IRT-5	Decreased	CD8^+^ T effector cells decreased	(16)
NOD.AIRE^−/−^	GF	No effect	Reduced inflammatory infiltrates in the retina	(14)
AIRE^GW/+^ LYN^−/−^ (C57BL/6)	Broad-spectrum antibiotics	No effect	LYN^−/−^ DCs present more IRBP to increase priming, not changed with microbiota	(15)

a Ampicillin, metronidazole, neomycin, vancomycin.

b Metronidazole and ciprofloxacin.

*GF, germ-free rederivation; SCFA, short chain fatty acid; IRBP, interphotoreceptor retinoid binding protein; IRT-5, a probiotic mix of Lactobacillus casei, L. acidophilus, L. reuteri, Bifidobacterium bifidum, and Streptococcus thermophiles*.

## Is Antigenic Mimicry by Microbiota an Etiologic Factor in Uveitis and in Autoimmunity in General?

IRBP is an immunologically privileged antigen in the eye, and is not expected to be expressed in the gut. To support the contention that the high frequency of Th17 cells in the gut LP is due to microbial stimuli, we introgressed the R161H TCR onto an IRBP^−/−^ background. Although R161H IRBP^−/−^ mice do not develop uveitis due to lack of the target antigen in their eyes, they exhibited similarly high frequency of Th17 cells in the gut compared to IRBP-sufficient R161H mice (5). These results indicated that endogenous IRBP is dispensable for the induction of Th17 cells in the gut, and supported the notion that IRBP-specific T cells in these mice are activated via non-cognate, likely microbial, antigens in the gut. Indeed, bacteria-rich intestinal contents activated R161H T cells *in vitro* and endowed them with the ability to transfer disease to naïve WT recipients. This activation was not seen when intestinal contents were heat-inactivated, or treated with Proteinase K, or when intestinal contents of GF mice were used, suggesting that proteinaceous components from microbiota are stimulating retina-specific T cells (5). We also tested the possible contribution of innate microbial stimuli, such as LPS or MTB extracts, as well as microbial superantigens, but none of these stimulants activated IRBP-specific T cells.

**Figure 3:**
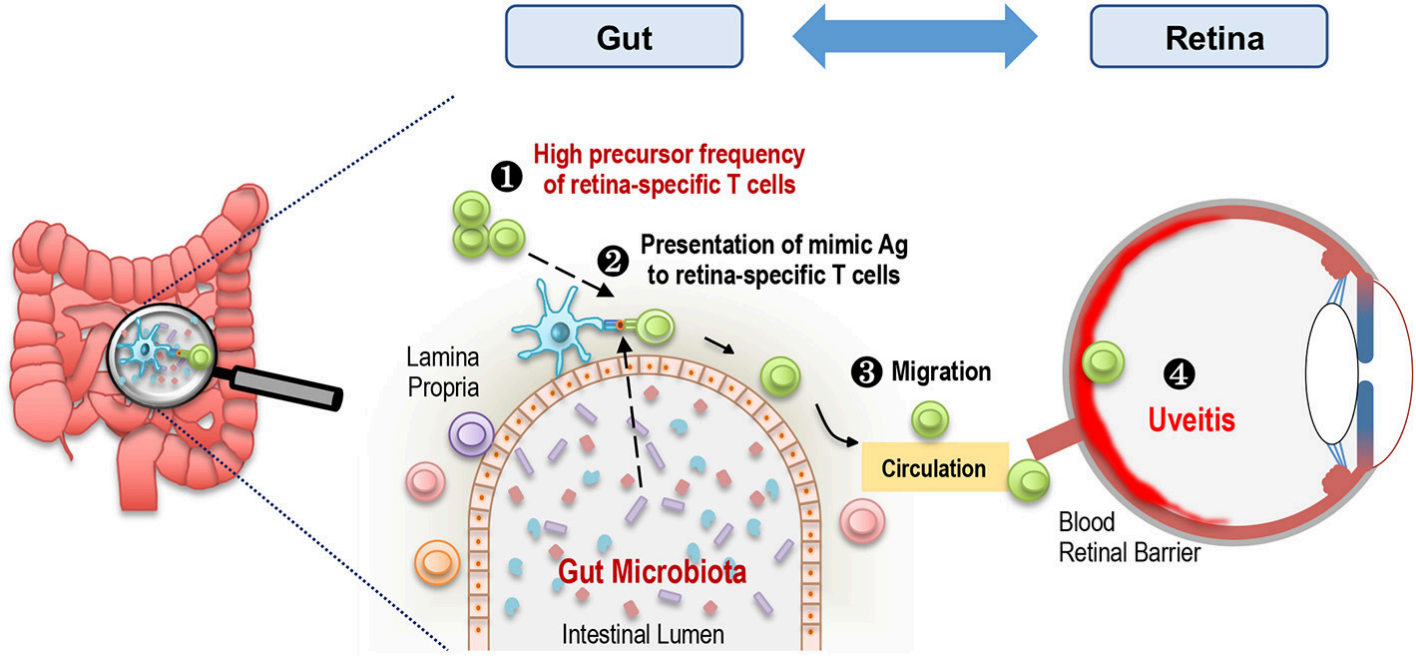
Microbiota-dependent activation of retina-specific autoreactive T cells triggers uveitis. A high frequency of retina-specific autoreactive T cells is present in the periphery including gut LP of R161H mice (1). Gut microbiota-derived products, which may mimic Ag to these T cells, are presented by antigen presenting cells (2). Retina-specific T cells become activated, migrate to the eye (3), and cross the blood retinal barrier to cause inflammation in the retina (4).

To further support the involvement of IRBP-specific clonotypic TCR in the activation of R161H T cells in the gut, we crossed R161H mice on the TCRα deficient background, to exclude possible participation of a second TCR, composed of an endogenous TCRα chain and the transgenic TCRβ chain. The data confirmed that activation of R161H T cells in the gut occurs through the clonotypic TCR by the criteria of phosphorylation of Zap70 and induction of the Th17 phenotype (5). These findings nevertheless do not negate a requirement for innate adjuvant effects, which can also come from microbiota. Figure 3 represents a graphic summary of how microbiota may contribute to spontaneous uveitis in this model. Although the putative antigenic mimic in the spontaneous uveitis model remains unidentified, a mimic of an islet-specific antigen responsible for type 1 diabetes (T1D) from Fusobacteria was recently reported using a diabetogenic TCR transgenic system in NOD mice (17). A high degree of similarity between a hypothetical protein of *Prevotella sp*. and human collagen epitopes was reported in periodontal disease (18). Similarly, putative microbial mimics were identified for two rheumatoid arthritis (RA)-associated autoantigens: N-acetylglucosamine-6-sulfatase (GNS) and filamin A (FLNA). Both are highly expressed in inflamed synovial tissue and share homologous T cell epitopes with Prevotella and other gut microbes. They are also the targets of specific T and B cell responses in RA patients (19) (Table 3). Thus, antigenic mimicry by commensals may be a more common and frequent trigger of autoimmune diseases than it is currently appreciated.

**Table 3:** Self-antigens and microbial mimicry in autoimmune diseases.

**Self-antigen**	**Tissue where self-antigen is expressed**	**Disease**	**Species**	**Microbe or mimicry**	**Reference**
IGRP	Islet	T1D	Mouse	*Leptotrichia goodfellowii* (Fusobacteria)	(17)
Collagen	Periodontal tissue	Periodontitis	Human	Prevotella sp. hypothetical protein	(18)
GNS	Synovial tissue	RA	Human	Prevotella and Prabacteroides	(19)
FLNA	Synovial tissue	RA	Human	Prevotella and Butyricimonas	(19)

*IGRP, islet-specific glucose-6-phosphatase catalytic subunit-related protein; GNS, N-acetylglucosamine-6-sulfatase; FLNA, filamin A; T1D, type 1 diabetes; RA, rheumatoid arthritis*.

## Microbial Products and Metabolites in the Intestine That Modulate Uveitis

Gut microbes are estimated to produce thousands of metabolites. Recent studies reveal that a class of microbial metabolites that are abundant in the gut regulate intestinal adaptive immune responses and promote health. Short chain fatty acids (SCFAs) are metabolites produced from fermentation by gut bacteria in the colon. Acetate, butyrate and propionate are produced by gut microbiota from high-fiber diet. SCFAs act by mechanisms including histone modification and G protein-coupled receptor (GPR) signaling (20). SCFAs induce and recruit Treg populations and are protective in animal disease models such as colitis (21). Interestingly, oral administration of SCFA, in particular propionate, in drinking water, starting 3 weeks prior to the immunization and continuing through the course of EAU, showed suppressive effects on disease progression in C57BL/6 mice, but not in B10.RIII mice (9). Propionate increased Tregs in the intestinal LP at early stage of EAU in C57BL/6 mice, promoted barrier functions and maintained the structural stability of the intestine. Other SCFA, acetate and butyrate which are also known to use the same GPRs as propionate and induce Tregs, appeared to have no effect on EAU. It is not clear why only propionate was effective in EAU in C57BL/6 mice. Multiple mechanisms may be involved in the mouse strain specific protection, including the early induction of Tregs in LP which, however, was not detected in B10.RIII mice. More studies are needed to better understand the effects of bacterial metabolites in uveitis.

## All the Way From the Gut to the Retina

The studies described in the sections above support a causative role of microbiota in triggering uveitis, but direct proof that autoreactive T cells that had become activated in the gut actually migrate and end up in the eye is still lacking. Transgenic mice that express the photoconvertible fluorescent protein Kaede (22) may provide an approach to address this question. The Kaede protein irreversibly changes its color from green to red following 405 nm violet light exposure. In theory, one could illuminate the cells in one tissue of Kaede transgenic mice and monitor the presence of photoconverted red cells in another tissue. This approach was taken by Morton et al. (23) to demonstrate that Th17 cells from the colon migrated into the spleen in the K/BxN arthritis model and by Krebs et al. showing trafficking of gut-derived lymphocytes to the kidney in a model of glomerulonephritis (24). Taking a similar approach, Nakamura et al. (9) used Kaede mice to photoconvert the lymphocytes in the colon and detect Kaede-red cells in the eye. Very tiny population (0.1% of total leukocytes) was detected by flow cytometry, supporting the interpretation that photoconverted cells can be detected in the eye. However, it must be kept in mind that this approach suffers from limitations that can confound data interpretation. The intestine has an abundant blood supply, therefore, not only cells within the tissue but also cells passing through the vasculature can also become photoconverted. Furthermore, because the retina itself is very highly vascularized, it is necessary to perfuse the ocular vasculature exhaustively to purge photoconverted blood-borne cells which have not entered the retina. Additional pitfalls are that photoconversion is not 100% (usually ~75%), only a segment of the gut can feasibly be exposed to the laser, and there is a limited period of time to detect the conversion. Therefore, extremely rigorous conditions and additional approaches and controls will be needed to provide irrefutable evidence that retina-specific T cells migrate from the gut to the eye to kick-start uveitis.

## Modulation of Gut Microbiota as a Therapeutic Approach for Uveitis

Probiotics, prebiotics and fecal transplants are gaining momentum as an approach to maintain intestinal health and treat some pathogenic conditions, which are mainly related to dysbiosis. Similar to the work by Nakamura et al. (9) who administered SCFA to modulate EAU, live probiotics were examined by Kim et al. for ability to modulate EAU in C57BL/6 mice (Table 2) and autoimmune dry eye disease in NOD.B10.*H2*^*b*^ mice, after pretreatment with broad-spectrum antibiotics for 5 days (16). The probiotic mix, IRT-5, consisting of *Lactobacillus casei, L. acidophilus, L. reuteri, Bifidobacterium bifidum*, and *Streptococcus thermophilus* had previously been demonstrated to treat EAE, and recently an active polysaccharide from *Bifidobacterium Bifidum* has been identified that has the ability to induce Tregs *in vitro and in vivo* (25, 26). Although therapeutic effects were observed in both eye disease models, the frequency of Tregs in the cervical lymph nodes of IRT-5 treated EAU mice was unexpectedly decreased, as was the frequency of effector CD8^+^ T cells, whereas numbers of effector CD4^+^ T cells were not affected (16). Given that CD4^+^ T cells are the pathogenic T cell population in IRBP-induced EAU and CD8 are dispensable, these results seem puzzling. Nevertheless, the beneficial effects of probiotics on clinical scores of EAU supports the notion that probiotics and their products should be explored as an approach to uveitis therapy. It is tempting to speculate that it may be plausible to use broad-spectrum antibiotics for a short period of time to reset the intestinal flora, and then to repopulate the gut with beneficial microbiota such as IRT-5 in the EAU model.

## Gut-Retina Axis vs. Gut-Brain Axis

The concept of gut-brain axis, a bidirectional communication between the brain and the gut, has emerged from research demonstrating that gut microbiota affects diseases in the CNS (27–29). In addition, accumulating evidence indicates that gut microbiome can also affect brain development and cognition (30, 31). The neural retina is a part of the CNS, as during embryonic development the retina and optic nerve originate as an outpocketing of the developing brain. The question is whether the gut-CNS concept applies to the retina independently of the brain, namely whether the gut-retina axis can be distinct from the gut-brain axis. A “gut-eye” axis was proposed in the study of secretory IgA in the eye-associated lymphoid tissue, showing that gut commensal microbiota plays a role in regulating ocular secretory IgA levels that protect ocular mucosal surface barrier function in Swiss Webster mice (32). Data discussed extensively in this review, presenting evidence that gut microbiota and their products trigger or modulate the autoimmune retinal disease, support the presence of such a gut-retina axis. A gut-retina axis was also proposed in a study of age-related macular degeneration (AMD). AMD is also the leading cause of blindness in developed countries, but in addition to the genetic or environmental factors, it is more associated with age, unlike uveitis. Using dietary glycemia-induced AMD mouse model, Rowan et al. demonstrated that changes in gut microbiota altered the production of metabolites, including serotonin, that are protective against AMD (33). These studies support the role of gut microbiota-derived products in the ocular disease but further study of bidirectional communication between the gut and the retina is needed to confirm and support the existence of a gut-retina axis.

## Human Uveitis and the Commensal Microbiome

The links between the gut microbiota and human health are well-established. Clinical studies support an association between changes in gut commensal microbiota (a.k.a. dysbiosis) and human autoimmune diseases including rheumatoid arthritis, spondyloarthritis, lupus, T1D, inflammatory bowel disease and multiple sclerosis (34, 35). The relative abundance of some bacterial taxa has been associated with the diseases in patients and in the corresponding animal models. One such example for uveitic diseases is the HLA-B27 transgenic rat model for spontaneous spondyloarthritis, analogous to human ankylosing spondylitis, which exhibited dysbiosis compared to healthy controls (36, 37). HLA-B27 is associated with acute anterior uveitis, which occurs in 30–40%, and ankylosing spondylitis patients have gut dysbiosis (38).

Although a link between inflammatory bowel disease and various forms of ocular inflammation, including uveitis, has long been appreciated (39), reports analyzing human gut microbiome in uveitis patients are sparse and cohort sizes are limited. Diseases studied include Behçet's syndrome, idiopathic uveitis and acute anterior uveitis (40–44) (Table 4). A recent study from Behçet's disease patients (44) revealed that fecal microbiota transplantation from patients into B10.RIII mice exacerbated EAU induced by active immunization. However, the gut microbiome in the mice that were colonized with patients' fecal material did not correspond with metagenomic species analysis in the donor patients. A coherent picture of taxa associated with different disease states has yet to emerge and needs further studies, including work in animal models to validate the findings in humans.

**Table 4:** Microbiota association in patients related to uveitis.

**Disease**	**Number of patients**	**Region**	**Results**	**Reference**
Behçet's disease	22^*^	Italy	Altered gut microbiota composition, reduction of butylate production	(40)
Behçet's disease	12 (5 had uveitis)	Japan	Altered gut microbiota composition, increased fecal secretory IgA	(41)
Behçet's disease	32	China	Altered gut microbiota composition, transplantation of fecal microbiota from patients exacerbated EAU	(44)
Autoimmune uveitis	13 (10 idiopathic, 3 VKH)	India	Altered gut microbiota composition	(42)
Acute anterior uveitis	38	China	No significant change in gut microbiota composition, altered fecal metabolic phenotype	(43)

* Presence of uveitis or eye inflammation was not mentioned.

*VKH, Vogt-Koyanagi-Harada disease*.

## Conclusions and Implications

As a part of the CNS, the retina plays a central role in our visual function, and therefore uveitis or any inflammatory insult in the retina is detrimental to quality of life. We reviewed recent progresses in gut microbiota in uveitis research in animal models and humans, which has been slow to advance, compared to the research on the gut-brain axis affecting diseases including MS, Parkinson's, and Alzheimer's disease. Associations between microbiota and uveitis are now established and identifications of causative or protective microbes are the important future steps to follow. The concept of an immune-driven gut-retina axis is actively being explored and existing data in animals and in humans raise the scepter of therapeutic approaches that might become possible through targeted manipulation of the microbiome.

## Author Contributions

RH made the outline of the manuscript. RH and RC wrote and finalized the manuscript.

### Conflict of Interest Statement

The authors declare that the research was conducted in the absence of any commercial or financial relationships that could be construed as a potential conflict of interest.
